# OpenMS-Simulator: an open-source software for theoretical tandem mass spectrum prediction

**DOI:** 10.1186/s12859-015-0540-1

**Published:** 2015-04-02

**Authors:** Yaojun Wang, Fei Yang, Peng Wu, Dongbo Bu, Shiwei Sun

**Affiliations:** 1 Key Lab of Intelligent Information Processing, Institute of Computing Technology, Chinese Academy of Sciences, 6, Kexueyuan South Road, Zhongguancun, Beijing, 100190 China; 2 University of Chinese Academy of Sciences, 19A, Yuquan Road, Beijing, 100049 China; 3 Institute of Biophysics, Chinese Academy of Sciences, 15, Datun Road, Chaoyang District, Beijing, 100101 China

**Keywords:** Mass spectrometry, Theoretical spectrum prediction, Peptide identification

## Abstract

**Background:**

Tandem mass spectrometry (MS/MS) acts as a key technique for peptide identification. The MS/MS-based peptide identification approaches can be categorized into two families, namely, *de novo* and database search. Both of the two types of approaches can benefit from an accurate prediction of theoretical spectrum. A theoretical spectrum consists of *m*/*z* and intensity of possibly occurring ions, which are estimated via simulating the spectrum generating process. Extensive researches have been conducted for theoretical spectrum prediction; however, the prediction methods suffer from low prediciton accuracy due to oversimplifications in the spectrum simulation process.

**Results:**

In the study, we present an open-source software package, called OpenMS-Simulator, to predict theoretical spectrum for a given peptide sequence. Based on the mobile-proton hypothesis for peptide fragmentation, OpenMS-Simulator trained a closed-form model for the intensity ratio of adjacent *y* ions, from which the whole theoretical spectrum can be constructed. On a collection of representative spectra datasets with annotated peptide sequences, experimental results suggest that OpenMS-Simulator can predict theoretical spectra with considerable accuracy. The study also presents an application of OpenMS-Simulator: the similarity between theoretical spectra and query spectra can be used to re-rank the peptide sequence reported by SEQUEST/X!Tandem.

**Conclusions:**

OpenMS-Simulator implements a novel model to predict theoretical spectrum for a given peptide sequence. Compared with existing theoretical spectrum prediction tools, say MassAnalyzer and MSSimulator, our method not only simplifies the computation process, but also improves the prediction accuracy.

Currently, OpenMS-Simulator supports the prediction of CID and HCD spectrum for peptides with double charges. The extension to cover more fragmentation models and support multiple-charged peptides remains as one of the future works.

**Electronic supplementary material:**

The online version of this article (doi:10.1186/s12859-015-0540-1) contains supplementary material, which is available to authorized users.

## Background

Tandem mass spectrometry (MS/MS) has been considered as an indispensable technique for high-throughput peptide identification and characterization in the field of proteomics [1]. Extensive researches have been conducted for peptide identification, and a collection of software packages have been developed, such as SEQUEST [2], MASCOT [3], X!Tandem [4], SCOPE [5], pFind [6], PEAKS DB [7], etc.

The MS/MS-based peptide identification approaches can be categorized into two families: (1) database searching approaches: for each peptide sequence in a database, the corresponding theoretical spectrum is predicted and compared against the query experimental spectrum. The most similar peptide-spectrum match (PSM) is reported as the final identification result. (2) *de novo* identification approaches: unlike the database search strategy, the *de novo* approach does not require a peptide sequence database as input. In essence, *de novo* approach can be treated as a search process working on a virtual peptide sequence database — the virtual database consists of all possible peptide sequences with the same precursor mass to the query experimental spectrum.

Accurate prediction of theoretical spectrum, including *m*/*z* and intensities of possibly occurring ions, is important to both database search and *de novo* identification approaches. Although theoretically possible, the accurate prediction of theoretical spectrum still remains a challenge due to the lack of deep understanding of the complex physical-chemical peptide fragmentation process during a MS/MS experiment. Therefore, most existing peptide identification tools employ an over-simplified model to simulate the peptide fragmentation process, leading to an inaccurate estimation of the ion intensities. Taking SEQUEST as an example, all *y*-ions are given a fixed intensity, regardless of the factors with substantial effects on the peptide fragmentation process, such as amino acid type and fragmentation sites, etc.

The relationship between peptide sequences and ion intensities has been studied to improve the accuracy of theoretical spectrum prediction [8-12]. A pioneer research of these works is the kinetic model used in MassAnalyzer, which simulates the peptide fragmentation pathways based on the “mobile proton” hypothesis. Another prediction method, MSSimulator, employs the support vector regression technique to predict the likelihood that an ion appears in a spectrum [13].

Based on the “mobile proton” peptide fragmentation model, we have proposed a novel theoretical spectrum prediction approach called MS-Simulator [14]. Unlike the existing approaches to predict ion intensities directly, MS-Simulator aims to predict the intensity ratio of every two adjacent *y*-ions. In brief, the intensity of a *y*-ion is determined by both near neighbouring amino acids and remote amino acids. The remote amino acids, however, were observed to have approximately equal effects on ion intensities *y*_*i*_ and *y*_*i*+1_, and thus can be canceled out when calculating intensity ratio $\frac {y_{i}}{y_{i+1}}$. In fact, only the two termini of peptides were employed in MS-Simulator to capture the effects of remote amino acids. Having acquired intensity ratios of all neighbouring ions, the whole spectrum can be easily constructed. It should be pointed out that unlike the kinetic model used in MassAnalyzer [15,16], the intensity ratio used by MS-Simulator has a closed-form; thus, the computation process is significantly simplified and the prediction accuracy is also considerably improved.

The study presents an open source package implementation of MS-Simulator called OpenMS-Simulator, which can be freely downloaded through our website.

## Implementation and results

OpenMS-Simulator package has four functionalities, namely, *theoretical spectrum prediction*, *PSM re-ranking*, *FDR analysis*, and *spectrum visualization*. These functionalities are briefly described as follows:

1. Theoretical spectrum prediction and spectrum visualization.
  OpenMS-Simulator takes peptide sequences as input and reports predicted theoretical spectra as output. A theoretical spectrum consists of *y*-ions and the corresponding isotopic derivatives. The current version of OpenMS-Simulator supports predicting theoretical spectra of both HCD (Higher-energy collisional dissociation) and CID (Collision-induced dissociation) types.
  OpenMS-Simulator provides the visualization of spectra by labelling all peaks with ion types. In addition, both theoretical spectrum and its experimental counterpart are shown in one frame to clearly display their similarity and difference. Pearson correlation coefficient (Pearson *CC*) is also calculated as a quantitative measure of the similarity (see Figure 1 for an example).

**Figure 1:**
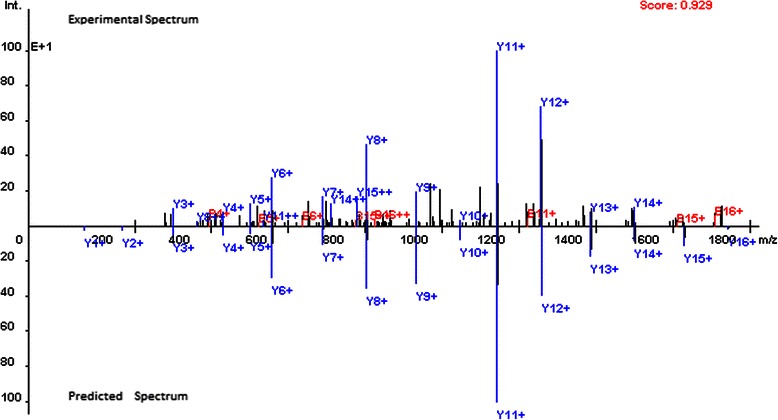
Comparison of the experiment spectrum and the predicted spectrum for peptide EIELEDPLENMGAQMVK (Pearson correlation coefficient = 0.929).
2. PSM re-ranking and FDR analysis.
  OpenMS-Simulator can also be used to re-rank the PSMs reported by SEQUEST or X!Tandem. More specifically, SEQUEST usually reports a peptide-spectrum match together with two scores, namely, *X*_*corr*_ and *Δ* *C*_*n*_, to measure the likelihood that the query spectrum is generated from the peptide. OpenMS-Simulator combines the two scores with Pearson *CC* to yield a new score, i.e. *X*_*corr*_+5∗*Δ* *C*_*n*_+5∗*C* *C*. For a PSM reported by X!Tandem, OpenMS-Simulator utilizes the score $\#SharedPeaks * \sqrt {S_{T}}*CC + S_{T}$, where *#* *S* *h* *a* *r* *e* *d* *P* *e* *a* *k* *s* denotes the number of peaks shared by experimental and predicted spectrum, and *S*_*T*_ refers to the score reported by X!Tandem. The new score is employed to re-rank PSMs reported by SEQUEST/X!Tandem.
  OpenMS-Simulator provides the functionality called FDR (False Discovery Rate) analysis to evaluate the performance of PSMs re-ranking. In particular, two FDR curves are drawn: one curve is calculated based on the original ranks given by SEQUEST/X!Tandem, and another curve is calculated according to the new score calculated by OpenMS-Simulator. This way, the improvement of re-ranking strategy can be intuitively demonstrated. The FDR was estimated using the decoy count method; that is:
  $$FDR=\frac{FP}{TP+FP} $$
  where *FP* denotes the number of false-positive peptide identifications, and *TP* denotes the number of true-positive identifications.

The theoretical spectrum prediction performance of OpenMS-Simulator is evaluated on the SwedCAD_7 T_LTQ-FT dataset (downloaded from http://www.bmms.uu.se/CAD/download.html). The dataset consists of 15,897 unmodified, doubly charged CID spectra together with highly confident peptide sequence annotations. On the dataset, the average Pearson *CC* between experimental and theoretical spectrum predicted by OpenMS-Simulator is as high as 0.890. In Figure 1, the theoretical spectrum predicted for peptide EIELEDPLENMGAQMVK is shown as an example.

We also evaluated OpenMS-Simulator by comparing with two other theoretical spectrum prediction models, namely MSSimulator that uses the support vector regression technique, and MassAnalyzer that uses a kinetic model (see Table 1). To make a fair comparison, we performed the evaluation on the dataset used by MSSimulator [13], which contains 15,324 doubly charged ion trap mass spectra. The prediction accuracy is measured by the similarity between experimental spectrum and theoretical prediction. Specifically, we used the following similarity measure suggested by MassAnalyzer [16].

$$similarity= \frac{\sum \sqrt{{I^{1}_{m}} {I^{2}_{m}}}}{\sqrt{\left(\sum {I^{1}_{m}}\right)\left(\sum {I^{2}_{m}}\right)}} $$

where ${I^{1}_{m}}$ and ${I^{2}_{m}}$ denote the intensities of the ions with *m*/*z* of *m* in the corresponding spectra.

**Table 1:** **Comparison of three theoretical spectrum prediction models**

**Similarity**	**Models**		
lr)2-4	**MSSimulator**	**MassAnalyzer**	**OpenMS-Simulator**
Mean	0.864	0.896	0.926
Variance	0.088	0.102	0.006

Dataset: 15,324 doubly charged spectra with peptide sequence annotations used by MSSimulator [13].

**Figure 2:**
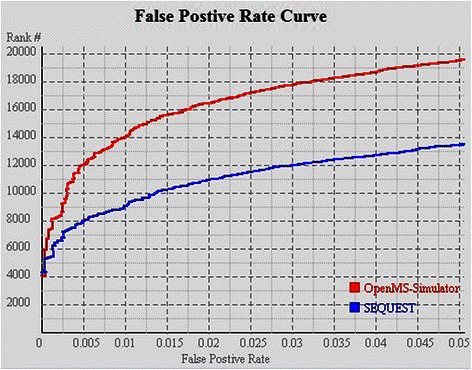
FDR curves of OpenMS-Simulator and SEQUEST on dataset PAe000350.

**Figure 3:**
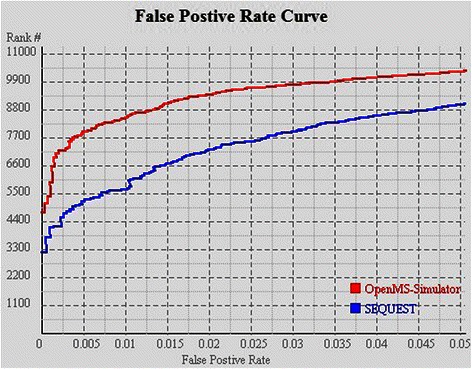
FDR curves of OpenMS-Simulator and SEQUEST on dataset PAe000351.

**Figure 4:**
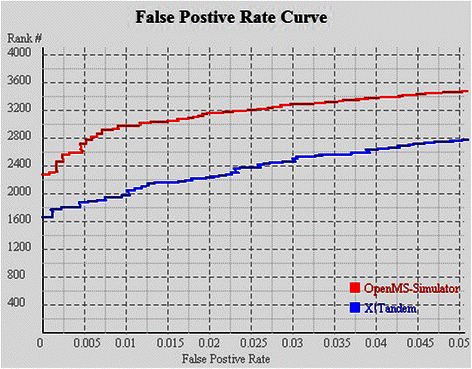
FDR curves of OpenMS-Simulator and X!Tandem on dataset PAe003641.

The re-ranking efficiency is evaluated on datasets PAe000350, PAe000351, and PAe003641 (downloaded from http://www.peptideatlas.org/repository). For each spectrum in PAe000350 or PAe000351, SEQUEST was executed to generate the most likely peptide sequence, and for spectra in PAe003641, X!Tandem was executed to give the peptide identification results. Subsequently the PSMs were re-ranked by running OpenMS-Simulator. FDR analysis suggests that by using the re-ranking strategy, the correctly identified PSM number can be significantly improved (see Figures 2, 3 and 4). In particular, when FDR is set as 0.005, SEQUEST can correctly identify 7,983 PSMs, while OpenMS-Simulator can correctly identify 12,001 PSMs (see Tables 2 and 3).

**Table 2:** **The number of correctly identified PSMs by OpenMS-Simulator and SEQUEST on dataset PAe000350 and PAe000351**

**Dataset**	**FDR = 0.005**		**FDR = 0.01**	
	**SEQUEST**	**OpenMS-**	**SEQUEST**	**OpenMS-**
		**Simulator**		**Simulator**
PAe000350	7,983	12,001	9,070	14,012
PAe000351	5,257	7,935	5,617	8,551

**Table 3:** **The number of correctly identified PSMs by OpenMS-Simulator and X!Tandem on dataset PAe003641**

**Dataset**	**FDR = 0.005**		**FDR = 0.01**	
	**X!Tandem**	**OpenMS-**	**X!Tandem**	**OpenMS-**
		**Simulator**		**Simulator**
PAe003641	1,872	2,721	1,985	2,993

## Model and parameters

Though intensive research has been conducted for peptide fragmentation, it is still not fully understood how a peptide fragments during mass spectrometry. Till now, the “mobile proton” hypothesis is one of the most widely-accepted explanations of the peptide fragmentation process, which consists of a collection of main peptide fragmentation pathways.

Based on the “mobile proton” hypothesis, OpenMS-Simulator employs a statistical model to predict intensity for possible ions, which extends our previous work MS-Simulator with several extensions and modifications. To avoid repetitions, only the extensions and modifications are listed as below:

1. The previous version of MS-Simulator supports prediction of theoretical CID spectrum only. OpenMS-Simulator has an extension to support prediction of HCD spectrum.
2. Compared with the previous version of MS-Simulator, more fragmentation pathways are taken into consideration in OpenMS-Simulator. Specifically, besides the common *b*_*x*_−*y*_*z*_ fragmentation pathway, the diketopiperazine pathway [8,17] was also incorporated in the model, enabling an accurate intensity prediction for *y*_*n*−1_ ion. The probability of the two pathways are denoted as *F*(*A*_*i*_) and *D*(*A*_*i*_,*i*), respectively. Thus, the Eq. 1 in MS-Simulator model was improved to be:
  $$\begin{aligned} \ln\frac{y_{i}}{y_{i+1}}= &\beta \times (E_{i}-E_{i+1}) + \ln(F(A_{i})+D(A_{i},i)) \\ - &\ln(F(A_{i+1})+D(A_{i+1},i+1)) \end{aligned} $$
3. Unlike MS-Simulator utilizing 5 consecutive neighbouring amino acids around the concerned *y*_*i*_ ion, only 4 neighbouring amino acids are used by OpenMS-Simulator to build prediction model. This way, the number of parameters is reduced with little influence on the prediction accuracy. The model parameters *Δ*(*x*,*d*) used in OpenMS-Simulator are summarized as follows:
  −2≤*d*≤1 for *x* being any amino acid except for LYS or ARG.
  −8≤*d*≤5 for *x* being LYS or ARG.
  3. 0≤*d*≤10 for *x* being *Cterm*.
  4. The effect of *Nterm* is divided into 5 levels according to *d*, i.e., *Δ*(*N* *t* *e* *r* *m*,*d*)≃*Δ* ^′^(*N* *t* *e* *r* *m*,*s*), where $s =\left \lceil \frac {5 \times d}{n} \right \rceil $.

The estimated parameters can be found in Additional file 1.

## Conclusions

We present an open source package OpenMS-Simulator implemented in Java language. OpenMS-Simulator can be used to accurately predict theoretical spectrum for a given peptide sequence. To show the performance of theoretical spectrum prediction, OpenMS-Simulator provides a functionality to re-rank PSMs reported by SEQUEST or X!Tandem. Experimental results suggest that the predicted theoretical spectrum help improve peptide identification.

## Availability and requirements

**Project name:** OpenMS-Simulator.**Project home page:** http://www.bioinfo.org.cn/OpenMS-Simulator.**Operating system:** Platform independent.**Programming language:** Java.**Other requirements:** Java 1.6 or higher.**License:** GNU GPL FreeBSD.**Any restrictions to use by non-academics:** licence needed.

## Additional file

Additional file 1
**Parameters estimation and long tables.**
