# Supplementary material for: OpenMS-Simulator: an open-source software for theoretical tandem mass spectrum prediction
Source: BMC Bioinformatics. 2015 Apr 2;16:110. doi: 10.1186/s12859-015-0540-1 (PMC4415337; doi:10.1186/s12859-015-0540-1)
Supplement: Additional file 1 — Parameters estimation and long tables. [file 12859_2015_540_MOESM1_ESM.pdf]

Supplement

Parameters estimation

The parameters of the model are optimized by the following training process over spectral data of known peptides: Since $\beta = 1/(R \times Reff)$ is a constant in one experiment, revised Eq.3 provides a linear equation for every two adjacent y-ions observed in the training data. By using a large number of training MS/MS spectra, thousands of linear equations are obtained on all parameters. The linear system is solved efficiently with the least-squares method to minimize the overall prediction error in the log intensity ratios. The optimized parameters trained from the CID data set are listed in Table S1, S2 and S3, and the optimized parameters trained from the HCD data set are listed in tables S4, S5 and S6.

To predict the y-ion intensities for a given peptide sequence, the intensity ratios between every two adjacent y-ions are first estimated using revised Eq.3 with optimized parameters. Having acquired all intensity ratios, the y-ion intensities can be easily calculated from the ratios. and the Eq.3 became:

$$
\begin{aligned}
\ln \frac{y_i}{y_{i+1}} =& \beta \times (\sum_{j=1}^{n} \Delta(A_j, j-i) + \Delta(Nterm, i) \\
&+\Delta(Cterm, n-i)) \\
&+ \ln(F(A_i) + D(A_i, i)) \\
&- \ln(F(A_{i+1}) + D(A_{i+1}, i+1)) \\
=& \beta \times (\sum_{j=1, j\neq i}^{n} \Delta(A_j, j-i) + \Delta^*(A_j) \\
&+\Delta(Nterm, i) + \Delta(Cterm, n-i)) \\
&+D^*(A_i, i) - D^*(A_{i+1}, i+1)
\end{aligned}
$$

Here, $\Delta^*(A_i) = \Delta(A_i, 0) + \ln F(A_i) - \ln F(A_{i+1})$, and $D^*(A_i, i) = \ln(1 + D(A_i, i)/F(A_i))$. $D^*(A_i, i)$ is introduced to describe the probability of 'diketopiperazine' pathway. It has been reported that *Cis-trans* isomerization seldom occurs if the fragment position is far from N-term; thus, $D^*(A_i, i)$ was set to 0 except for the 3 closest neighbours of N-term.

Table 1: Optimized parameters of OpenMS-Simulator for CID spectrum: $D^*(A,i)$ $(i=0,1,2)$, and $\beta\cdot\Delta\left(x,d\right)$ for the four amino acids nearby the concerned y_i and y_{i+1} ions

Residue	-2	-1	0	1	$D^*(A,0)$	$D^*(A,1)$	$D^*(A,2)$
ALA	-0.261	0.130	0.033	-0.081	2.600	-0.919	0.010
CYS	-0.204	-0.283	-0.452	-0.113	3.192	-0.524	0.277
ASP	0.111	-0.026	0.127	-0.266	0.535	-0.865	0.058
GLU	-0.002	-0.172	0.314	-0.335	1.572	-0.899	0.070
PHE	-0.423	-0.043	-0.066	0.113	2.871	-0.816	0.120
GLY	-0.114	1.075	-1.451	0.087	2.309	-0.829	0.115
HIS	-0.821	-0.225	-0.048	0.318	1.666	-0.913	0.176
ILE	-0.400	-0.518	0.766	-0.077	2.899	-0.925	0.103
LYS	-0.509	-0.454	0.590	-0.065	0.633	-0.854	0.276
LEU	-0.484	-0.128	0.568	-0.057	2.661	-0.937	0.074
MET	-0.341	-0.069	0.270	-0.200	1.843	-0.996	0.050
ASN	-0.177	0.540	-0.431	-0.198	2.089	-0.818	0.181
PRO	-0.057	0.604	-2.977	0.919	1.453	-0.396	0.526
GLN	-0.212	-0.191	0.483	-0.370	1.934	-0.885	0.155
ARG	0.068	-0.163	-0.184	-0.299	0.214	-0.863	0.626
SER	-0.226	0.542	-0.698	0.025	2.713	-0.956	0.037
THR	-0.349	0.316	-0.235	-0.053	3.051	-1.033	0.096
VAL	-0.400	-0.515	0.734	-0.057	2.893	-0.922	0.075
TRP	-0.534	-0.243	-0.045	0.210	2.639	-0.641	0.043
TYR	-0.439	-0.163	-0.076	0.093	2.921	-0.750	0.156

Table 2: Optimized parameters of OpenMS-Simulator for CID spectrum: $\beta\cdot\Delta(\texttt{LYS},d)$ and $\beta\cdot\Delta(\texttt{ARG},d)$

d	-8	-7	-6	-5	-4	-3	1	2	3	4	5
LYS	0.137	-0.276	-0.134	-0.163	-0.157	-0.057	0	0.021	-0.023	-0.173	-0.143
ARG	0.428	-0.337	-0.013	0.221	-0.087	-0.252	-1.306	0.061	0.058	0.083	0.093

Table 3: Optimized parameters of OpenMS-Simulator for CID spectrum: $\beta\cdot\Delta'(\texttt{Nterm},s)$ and $\beta\cdot\Delta(\texttt{Cterm},d)$

d or s	1	2	3	4	5	6	7	8	9	10
$\Delta(\texttt{Cterm},d)$	0	-1.306	-1.167	-0.971	-0.618	-0.531	-0.388	-0.269	-0.179	-0.086
$\Delta'(\texttt{Nterm},s)$	0.067	-0.323	-0.531	-0.327	-0.154	0	0	0	0	0

Table 4: Optimized parameters of OpenMS-Simulator for HCD spectrum: $D^*(A,i)$ $(i=0,1,2)$, and $\beta \cdot \Delta(x,d)$ for the four amino acids nearby the concerned y_i and y_{i+1} ions

Residue	-2	-1	0	1	$D^*(A,0)$	$D^*(A,1)$	$D^*(A,2)$
ALA	-0.646	2.457	-0.127	0.502	1.624	-0.540	0.112
CYS	-0.567	2.442	-0.563	0.503	1.185	-0.625	0.267
ASP	-0.354	2.082	0.218	0.268	1.674	-0.596	-0.006
GLU	-0.475	2.127	0.334	0.120	1.847	-0.595	0.060
PHE	-0.752	2.315	-0.132	0.667	1.056	-0.464	0.234
GLY	-0.531	3.382	-1.426	0.569	1.530	-0.693	0.116
HIS	-1.019	2.096	-0.427	0.948	0.222	-1.006	-0.004
ILE	-0.769	1.842	0.687	0.452	1.045	-0.503	0.184
LYS	-0.924	1.928	-0.003	0.525	-0.138	-0.806	0.030
LEU	-0.815	2.156	0.414	0.481	1.019	-0.483	0.180
MET	-0.530	2.250	0.216	0.361	0.815	-0.561	0.202
ASN	-0.509	2.732	-0.445	0.335	1.359	-0.536	0.166
PRO	-0.677	2.932	-2.284	1.810	-0.551	-0.577	0.532
GLN	-0.595	2.164	0.382	0.078	1.356	-0.533	0.221
ARG	0.090	2.849	-0.488	-0.332	-1.221	-1.477	0.042
SER	-0.562	2.831	-0.796	0.584	1.693	-0.621	0.083
THR	-0.677	2.575	-0.340	0.547	1.627	-0.550	0.191
VAL	-0.721	1.876	0.606	0.448	1.257	-0.499	0.155
TRP	-0.828	2.135	-0.044	0.817	0.495	-0.460	0.315
TYR	-0.768	2.270	-0.115	0.684	0.982	-0.447	0.227

Table 5: Optimized parameters of OpenMS-Simulator for HCD spectrum: $\beta \cdot \Delta(\texttt{LYS}, d)$ and $\beta \cdot \Delta(\texttt{ARG}, d)$

d	-8	-7	-6	-5	-4	-3	1	2	3	4	5
LYS	0.112	0.159	0.119	0.138	0.058	0.012	-0.262	-0.322	-0.152	-0.109	-0.240
ARG	0.331	0.533	0.508	0.631	0.518	0.561	0.596	-0.873	-0.580	-0.358	-0.250

Table 6: Optimized parameters of OpenMS-Simulator for HCD spectrum: $\beta \cdot \Delta'(\texttt{Nterm}, s)$ and $\beta \cdot \Delta(\texttt{Cterm}, d)$

d or s	1	2	3	4	5	6	7	8	9	10
$\Delta(\texttt{Cterm}, d)$	0	0.258	-0.015	-0.349	-0.373	-0.315	-0.423	-0.343	-0.199	-0.084
$\Delta'(\texttt{Nterm}, d)$	-0.241	-0.513	-0.492	-0.298	-0.268	-0.344	0	0	0	0
